# Elevated Hair Cortisol Levels among Heroin Addicts on Current Methadone Maintenance Compared to Controls

**DOI:** 10.1371/journal.pone.0150729

**Published:** 2016-03-24

**Authors:** Jin Yang, Jifeng Li, Guanyi Xu, Jing Zhang, Zheng Chen, Zuhong Lu, Huihua Deng

**Affiliations:** 1 Key Laboratory of Child Development and Learning Science (Southeast University), Ministry of Education, and Institute of Child Development and Education, Research Center for Learning Science, Southeast University, Nanjing, 210096, China; 2 Center of Methadone Maintenance Treatment, Baixia District Hospital, Nanjing, 210004, China; 3 Key Laboratory of Environmental Medicine Engineering, Ministry of Education, School of Public Health, Southeast University, Nanjing, 210009, China; Medical University of Vienna, AUSTRIA

## Abstract

Whether methadone maintenance treatment (MMT) can improve the basal function of the hypothalamic–pituitary–adrenal (HPA) axis, which is suppressed by long-term heroin consumption, is a matter of debate. The stress state and depression and anxiety symptoms may affect the basal activity of the HPA axis in MMT patients. However, the effect of psychological factors on HPA activity was not simultaneously controlled in previous studies. This study investigated differences in HPA basal activity between MMT patients and controls using psychological variables as covariates. The participants included 52 MMT patients and 41 age-matched, non-heroin-dependent controls. Psychological states were self-reported with the Perceived Stress Scale, Self-Rating Depression Scale and Self-Rating Anxiety Scale. The hair cortisol level was adopted as a biomarker of HPA basal activity and was determined with liquid chromatography tandem mass spectrometry. The results revealed that MMT patients had significantly higher hair cortisol levels than the controls (*p*<0.05), but the difference was not significant (*p*>0.05) when the perceived stress, depression and anxiety scores were used as covariates. We concluded that patients with long-term MMT showed higher basal activity of the HPA axis. The high chronic stress state and increase in depression and anxiety symptoms may mask the suppression effect of methadone on the HPA activity.

## Introduction

Long-term heroin use not only produces harmful effects to addicts’ physiological and mental health but also results in a series of pathological changes in the brain function and structure because heroin dependence is a chronic relapsing brain disorder[1]. Heroin users usually exhibit atypical circadian patterns of the secretion of the hormones of the hypothalamic-pituitary-adrenal (HPA) axis and adrenal insufficiency due to the impairment of the HPA axis[2,3]. Generally, heroin use alone is associated with the suppression of the basal activity of the HPA axis, resulting in a lower than normal cortisol level[4–6].

Methadone is a synthetic opioid that is a full agonist of the μ-opioid receptors[7]. Methadone maintenance treatment (MMT) results in excellent treatment retention and favorable outcomes among heroin-dependent patients [8], For instance, MMT relieves heroin withdrawal reactions and attenuates the euphorigenic experiences of continued heroin use, thereby effectively curbing heroin craving [7]. MMT also significantly improves the life functioning of heroin-dependent patients, decreases their dosage of heroin use, reduces their criminal behaviors and the risk of relapse, and allows them to refrain from the practice of heroin use, thereby diminishing the risk of infection with human immunodeficiency virus due to needle sharing during heroin use[9]. However, researchers have questioned whether MMT can improve the function of the HPA axis impaired by heroin dependence and induce the recovery of the basal cortisol level to the normal level. Some researchers have proposed that MMT tends to normalize the function of the HPA axis among heroin addicts [10] despite the suppression of the function of the HPA axis by methadone, resulting in lower basal cortisol secretion based on theoretical pharmacotherapy [1]. The results of the existing empirical studies on the cortisol levels of MMT patients are inconsistent, with some studies reporting an increase relative to healthy controls [3,4,11–15], a decrease[4,16,17] and others reporting no change [18]. To date, the reasons for the inconsistency remain unclear.

One possible explanation might be that the biological index used in previous studies showed methodological limitations in the proper assessment of the basal activity of the HPA axis[19]. Most previous studies utilized cortisol levels in the plasma, saliva and urine as the biomarkers to assess the basal cortisol level[11,13,17]. These biomarkers are susceptible to circadian rhythms and the incidents that occurred prior to sampling. Recently, endogenous cortisol levels in human hair were suggested to overcome the limitations and were proven to be a retrospective index of cumulative cortisol exposure over periods of up to 6 months[19]. Another explanation might be that the participants in previous studies were in different stages of the detoxification reaction. For example, heroin addicts undergoing acute withdrawal syndrome might be in the early stage of detoxification [4,20,21], whereas abstinent addicts with long-term MMT might be in the late stage of detoxification [14,20,21]. Heroin addicts in the early detoxification stage may be in a high stress state [21] because they are undergoing acute withdrawal syndrome and showing strong biological stress responses, such as hypercortisolism[4] and hyper-responsivity of cortisol[10,12,13,15,16,22]. Additionally, the basal activity of the HPA axis in MMT patients may be influenced by negative emotions; for example, MMT patients with more depression symptoms exhibit higher cortisol levels [6]. Moreover, there may be synergistic effects of both psychological factors (i.e., stress state/or depression and anxiety symptoms) and MMT on the function of the HPA axis. Therefore, it is essential to control the potential influences of psychological factors when investigating the effect of long-term methadone maintenance on the function of the HPA axis in MMT patients. However, previous empirical studies did not simultaneously examine the effects of the stress state and depression and anxiety symptoms.

This study selected the hair cortisol level as a biomarker of the basal activity of the HPA axis. Participants selected in this study were patients who were currently receiving methadone maintenance treatment for a period longer than 3 months because heroin addicts who received MMT for less than 3 months might remain in a high stress state and exhibit symptoms of acute withdrawal syndrome[21]. The present study examined whether there was a significant difference in hair cortisol levels between MMT patients and non-heroin-dependent healthy controls when the stress state and depression and anxiety symptoms were used as covariates. Because previous studies demonstrated that the hair cortisol level was an endogenous biomarker in healthy participants[23,24], we needed to confirm whether the hair cortisol level was also an endogenous biomarker for MMT patients. Finally, we validated our findings by examining the association between urinary cortisol and hair cortisol among MMT patients.

## Methods

### Participants

The participants were male heroin addicts on current methadone maintenance treatment recruited from a public center for MMT service of outpatients in Nanjing, China; non-heroin-dependent participants were included as controls. All participants provided written informed consent prior to inclusion. The present study followed the Declaration of Helsinki and was approved by the Health Science Research Ethics Board of Southeast University.

The inclusion criteria for the MMT participants were age older than 18 years; history of heroin dependence that met the DSM-IV criteria and included documented use of greater than 1 year; on current methadone maintenance; and steady-dose methadone maintenance for at least 3 months. The exclusion criteria included: 1) use of other drug except methadone over the past 3 months, including heroin, cocaine, cannabis, benzodiazepines and amphetamines; 2) alcohol addiction; 3) body mass index (BMI) >28 kg/m^2^; 4) mental disorders, including post-trauma stress disorder (PTSD); 5) HIV infection; 6) chronic diseases, such as hepatic, renal, cardiovascular and pulmonary diseases; 7) medication including the use of glucocorticoid drugs or antidepressants over the past 3 months; 8) dyed, perm, bleached or shorter hair (< 1 cm); and 9) smoking more than ten cigarettes per day. Exclusion criteria for the controls were 1) illicit drug use; 2) alcohol addiction; 3) smoking more than ten cigarettes per day; 4) body mass index (BMI) >28 kg/m^2^; 5) PTSD and mental disorders; 6) chronic diseases, such as hepatic, renal, cardiovascular and pulmonary diseases; 7) medication, including the use of glucocorticoid drugs or antidepressants over the past 3 months; and 8) dyed, perm, bleached or shorter hair (< 1 cm).

The MMT group initially included 263 men. One hundred and forty-five heroin addicts were excluded due to polydrug use and/or glucocorticoid drug use, HIV medication or mental disorders, and 23 heroin addicts were excluded due to a body mass index (BMI) > 28 kg/m^2^ and dyed, perm, bleached or shorter hair (< 1 cm). Ninety-five heroin addicts provided hair samples. Among them, 43 participants were excluded because of a hair weight less than 20 mg and a history of smoking. Finally, a total of 52 heroin addicts participated in the present study for hair cortisol analysis. These participants went to the MMT center every day after a three-month detoxification treatment and received an oral administration of a methadone dose of 5–80 mg/day because methadone has a half-life of 24–36 h[7]. The participants underwent periodic urine drug testing for heroin, cocaine, cannabis, benzodiazepines and amphetamines (enzyme-immunoassay) every month. Of these, 29 participants provided their overnight urine for the examination of the association between the urinary and hair cortisol levels. The control group included 41 non-addicted healthy male adults over 18 years of age recruited from a common residents’ community in Nanjing, China.

### Measurements and survey

Chronic stress was assessed using the 14-item Perceived Stress Scale (PSS) developed by Cohen et al.[25]. The PSS is a 4-point Likert scale. The raw total score ranges from 14 to 56, with higher scores indicating higher perceived stress. Anxiety and depression were assessed using the Self-Rating Anxiety Scale (SAS) (Zung, 1971) and the Self-Rating Depression Scale (SDS) (Zung, 1965), respectively. The 20-item SAS measures the anxiety tendency of subjective feelings based on the subjects’ self-report and provides a raw total score of 20 to 80. Raw scores less than 40 are assessed as normal (non-anxiety), 40−47 as mild anxiety levels, 48–55 as moderate anxiety levels, 56–63 as severe anxiety levels, and above 64 as extreme anxiety levels (Zung, 1971). The SDS is a short, self-administered survey to quantify the depressed status of a patient. There are 20 items on the scale that rate the affective, psychological and somatic symptoms associated with depression. Raw scores of the SDS range from 20 to 80. The raw scores are cataloged into four levels: 20–39 (normal range); 40–47 (mildly depressed); 48–55 (moderately depressed); and 56 and above (severely depressed) (Zung, 1965). These measurements have been widely used in Chinese populations and show good validity and reliability.

All participants were asked to indicate how they felt during the past month to assess their psychological status over the period that corresponded to the time period of 1-cm hair growth. Additionally, participants in the MMT group provided demographic data and other information, including employment status and durations of heroin addition and methadone maintenance treatment. The duration of heroin consumption referred to the time period from the beginning date of heroin consumption to detoxification.

### Collection of hair and urine

This study followed the procedure proposed by the Society of Hair Testing to collect hair samples longer than 1 cm in the posterior vertex region. Because the average hair growth rate is 1 cm per month[26], the 1-cm hair segment closest to the scalp would reflect the cortisol status during the last one-month period. Because 1–3 mm of the hair strands were deeply embedded in the skin and the 1–2 mm hair strands closest to the scalp cannot be completely cut with scissors, hair collection was performed two weeks after the completion of the survey data collection to ensure that the cortisol content of the collected hair sample reflected the psychological state of the participants during the survey data collection time point. The collected hair samples were stored at −20°C prior to analysis. The 1-cm segments closest to the scalp were used in the following experiments. Additionally, the collection of urine samples was performed prior to survey data collection. Participants provided their overnight urine samples after they wakened in the morning; the urine samples were stored at −20°C prior to analysis.

### Cortisol measurements

The cortisol content in the hair samples was analyzed with liquid chromatography tandem mass spectrometry (LC-MS/MS) (3200Qtrap, ABI, USA) and was described in detail elsewhere[27]. Briefly, the hair samples were ultrasonically washed with 5 ml of methanol for 2 min at room temperature to remove contaminants and non-blood-borne cortisol. After washing, the hair strands were milled by a ball mill (MM 400, Retsch, Germany) for 4 min at 30 Hz. A total of 20 mg of hair powder was incubated at 40°C for 14 h in 1 ml of methanol in the presence of 2.5 ng of cortisol-d4. Then, the incubation solution was separated by centrifugation at 12,000 rpm for 10 min. The supernatant was transferred to a dry tube and evaporated to dryness by N_2_ at 50°C. The dry residue was resuspended with 50 μl of methanol and then diluted to 1 ml in water. The diluted solution was extracted with an SPE C_18_ column activated sequentially by 3 ml of methanol and 3 ml of water. The deposit on the SPE C_18_ column was rinsed with 3 ml of water, dried for 30 min and eluted with 1 ml of methanol. The eluate was evaporated to dryness in pure N_2_ at 50°C and redissolved in 50 μl of methanol for LC-MS/MS analysis.

Urine samples were diluted 20-fold to effectively reduce the matrix effect. The treatment of urine samples was briefly described as follows. Urine samples were vortex-mixed uniformly for 1 min after they were thawed from −20°C. After centrifuging at 12,000 rpm for 5 min, 100 µl of the supernatant was transferred and diluted with 400 µl of water. After vortex-mixing for 1 min, 100 µl of the diluted urine solution was transferred to a dry tube, and 300 µl of water and 900 µl of ethyl acetate with 2.5 ng cortisol-d4 were added. The mixed solution was centrifuged at 12,000 rpm for 1 min. Then, 800 µl of the supernatant was transferred into another dry tube and evaporated to dryness in pure N_2_ at 50°C. The dry residue was redissolved in 100 μl of methanol for LC-MS/MS analysis.

The validation of the present LC-MS/MS method was achieved with the standard cortisol concentration of 50 pg/mg spiked with 20 mg of a blank hair matrix or 20 ng/ml of cortisol spiked with 100 µl of a blank urine matrix. The detection limit at the signal-to-noise ratio of 3 was 0.5 pg/mg and 0.2 ng/ml for the hair and urine analyses, respectively. The recovery (n = 5) was 97.0±5.1 and 98.1±4.8% for the hair and urine analyses, respectively. The intra-day and inter-day coefficients of variation (n = 5) were 6.0 and 5.7% for the hair analysis and 4.5 and 6.7% for the urine analysis, respectively.

### Statistical analysis

The education level was cataloged as elementary school and below (EL1), junior high school (EL2), senior high school (EL3) and college and university and over (EL4). The education level subgroup with less than 10 participants was excluded when examining the effect of the education level on the demographics, psychological variables, and hair cortisol contents.

Data (data in S1 Dataset)analysis was performed with the statistical package SPSS 16.0 for Windows. Statistical significance was accepted as p<0.05. A one-sample Shapiro-Wilks test was used to examine normally distributed data. Non-normally distributed data were expressed as the median and range, and normally distributed data were expressed as *M*±3*SD*. where *M* is the mean and *SD* is the standard deviation. Non-normally distributed data were log-transformed for the t-test, univariate analysis of variance (univariate ANOVA), Pearson correlation analysis and hierarchical multiple regression analysis because log-transformation effectively reduced the skewness and kurtosis. The Chi-square test was conducted for the comparison of the education level and prevalence of depression and anxiety between the MMT and control groups. A Mann-Whitney U test was performed for two independent samples for the comparison of non-normally distributed data (e.g., hair cortisol contents) between the MMT and control groups for the age, PSS, SDS and SAS scores, and the hair cortisol contents between the employment and unemployment subgroups and between the education level subgroups because there were less than 30 patients in each group. The *t*-test for two independent samples was conducted to compare normally distributed data (e.g., age, PSS, SDS and SAS scores, and log-transformed hair cortisol contents). Univariate ANOVA was conducted to compare the log-transformed hair cortisol contents between the MMT and control groups when demographical variables and psychological characteristics showing group differences were used as the covariates. Pearson correlation analysis was performed to examine the association of the log-transformed hair cortisol contents with the log-transformed urinary cortisol, age, PSS, SDS and SAS scores, MMT administration and heroin consumption. Hierarchical multiple regression analysis was performed to examine the durations of MMT administration and heroin consumption as the predictors of the log-transformed hair cortisol contents.

## Results

Two participants were excluded from the urine analysis because their urine samples were not frozen at -20°C. Among the remaining 27 MMT patients, the cortisol contents in their hair and overnight urine samples were non-normally distributed (*p*<0.05) but became normally distributed after log-transformation (*p*>0.05). The cortisol contents in the overnight urine samples (50.5±44.5 ng/ml) were significantly and positively correlated with the hair cortisol contents (12.8±13.3 pg/mg) (*r* = 0.481, *p* = 0.011).

The SDS, SAS and PSS scores in both the MMT (*n* = 52) and control groups (*n* = 41) were normally distributed (*p*s>0.120). The hair cortisol contents were non-normally distributed (*p*s<0.001) but became normally distributed (*p*s>0.699) after log-transformation. S1 Table (Table A in S1 Table) provided the demographic data and psychological states of the MMT patients and controls. There were no significant differences in age, PSS, SDS and SAS scores, and hair cortisol contents between the EL3 (*n* = 19) and EL4 (*n* = 14) subgroups among the controls (*p*s>0.186), and between the EL2 (*n* = 29) and EL3 (*n* = 17) subgroups, employment (*n* = 11) and unemployment (*n* = 33) subgroups, whole family (*n* = 33) and broken family (*n* = 19) subgroups, and the married (*n* = 27) and divorced (*n* = 20) subgroups among the MMT patients (*p*s>0.160).

As listed in S1 Table (Table A in S1 Table), there was no significant difference in age between the MMT participants (*p* = 0.464) relative to the controls, but the MMT participants did have a significantly lower education level (*p*<0.001), significantly higher SDS and SAS scores (*p*s<0.001) and a higher prevalence of depression and anxiety than the controls (*p*s<0.001). Additionally, the MMT patients had significantly higher PSS scores than the controls (27.5±6.6 *vs* 18.1±6.3, *t*_91_ = 7.007, *p*<0.001). Notably, the MMT patients exhibited significantly higher hair cortisol contents than the controls (*U* test: 10.0, 0.96–68.5 pg/mg *vs* 6.45, 1.43–28.0 pg/mg, *Z* = -1.997, *p* = 0.046 and *t*-test: 14.8±15.2 *vs* 8.5±6.1 pg/mg, *t*_90.232_ = 2.051, *p* = 0.043 for log-transformed data). Furthermore, the univariate ANOVA revealed that the MMT patients exhibited significantly higher hair cortisol contents than the controls (*F*_1, 87_ = 4.369, *η*_p_^2^ = 0.048, *p* = 0.040), although the inclusion of the age and education levels as covariates did not have significant influences (*p* = 0.125 and *p* = 0.875). However, the MMT patients exhibited higher hair cortisol contents than the controls with marginal significance (*F*_1, 84_ = 3.452, *η*_p_^2^ = 0.039, *p* = 0.067) when the PSS, SDS and SAS scores together with the age and education level were used as covariates. The covariates showed no significant influences (*p*s>0.134).

The log-transformed hair cortisol contents of the MMT patients were not significantly correlated with their age (n = 52, *r* = 0.128, *p* = 0.365), PSS (n = 52, *r* = -0.162, *p* = 0.251), SDS (n = 52, *r* = -0.176, *p* = 0.211), SAS (n = 52, *r* = -0.086, *p* = 0.544), durations of MMT administration and heroin consumption (n = 51, *r* = -0.228, *p* = 0.108 and n = 51, *r* = 0.004, *p* = 0.979). Furthermore, the hierarchical multiple regression analysis revealed that the durations of MMT administration and heroin consumption did not significantly predict the log-transformed hair cortisol contents (*p*s>0.05; S2 Table) (Table B in S2 Table) when the age, PSS, SDS and SAS scores were controlled.

## Discussion

This study found that the hair cortisol content showed a significant positive association with the urinary cortisol content among MMT patients. This finding was consistent with the previous finding that hair cortisol showed a significant correlation with the cortisol in 24-h urine (*r* = 0.33, *p*<0.05) among healthy participants[23]. Urinary cortisol has been demonstrated to be a physiological indicator of the HPA activity and stress state. Therefore, the present finding indicated that hair cortisol in MMT patents could be used as an endogenous physiological indicator of the basal activity of the HPA axis and stress state.

This study also found that MMT patients showed significantly higher perceived stress levels than non-heroin-dependent healthy controls. As shown above, the present patients were on current methadone maintenance and had received MMT for more than 3 months (mean duration: 33.8±26.2 months). Visiting the MMT center for long-term oral administration of methadone every day might be a significant stressful event for MMT patients. Moreover, most of the present MMT patients experienced other stressful events (S1 Table), such as lower education levels, unemployment, a broken family and divorce. Additionally, drug addicts face discrimination in China even if they withdrew and would not consume heroin[28]. This factor meant that it would be difficult for MMT patients to obtain effective support from their family and society. These stressful events induced a high chronic stress state in MMT patients and caused them to experience more negative emotions than the controls, such as more depression and anxiety symptoms.

Importantly, this study found that patients with long-term MMT showed significantly higher hair cortisol levels than the controls. Our finding was consistent with the results in previous studies where serum cortisol and salivary cortisol were used as biomarkers[3,4,11,12,14,15,29]. To the best of our knowledge, our study is the first report on hair cortisol levels of heroin-dependent patients with long-term methadone maintenance treatment.

This study further found that there was no significant difference in the hair cortisol levels between the MMT patients and the controls when the chronic stress state, depression, and anxiety symptoms were used as covariates together with the age and education level. Because heroin dependence impairs the function of the HPA axis and gives rise to a lower cortisol level than the normal level [4–6], the present results indicate that long-term methadone administration may recover the deregulated hormone secretion of heroin-dependent patients to the normal level. Thus, long-term methadone maintenance might to some extent improve the activity of the HPA axis and even the brain function impaired by heroin dependence. This result was consistent with the hypothesis proposed by Kreek et al. (2000). However, it was contrary to methadone’s pharmacological mechanism, where methadone suppresses the function of the HPA axis [1], resulting in lower basal cortisol secretion relative to the controls. This discrepancy might be due to the masking of the suppressive effect of methadone on the function of the HPA axis by the following factors. First, methadone’s suppressive effect might be masked by the elevation effect of the high chronic stress state and the presence of more negative emotions. As discussed above, the present MMT patients on current methadone maintenance were in a higher chronic stress state and experienced more depression and anxiety symptoms relative to the non-heroin-dependent healthy controls. Indeed, chronic stress activates an individual’s HPA activity and induces a significant elevation in the cortisol levels. Recent studies consistently demonstrated the significant increase of hair cortisol following systemic exposure to a series of chronic stresses [30–36]. Higher hair cortisol levels were closely associated with more depression and anxiety symptoms[36–38]. A previous study found that MMT patients exhibited a positive association of the cortisol level with depression symptoms [6]. Thus, the higher chronic stress state and increase in depression and anxiety symptoms might elevate the basal activity of the HPA axis in patients on current methadone maintenance. Notably, the present study found that the higher hair cortisol contents in the MMT patients relative to the controls became marginally significant after the chronic stress state and depression and anxiety symptoms were used as covariates. This finding implied that these psychological factors might elevate the basal function of the HPA axis. However, the psychological factors used as covariates did not have significant influences in the present study. Therefore, the psychological factors may be confounders, but the extent is still under debate. Second, the basal cortisol level might be affected by previous traumatic events, particularly early adverse traumatic experiences. Previous studies demonstrated that adverse traumatic experiences might elevate the HPA-axis activity and cortisol secretion[34,36], while other studies showed an opposite effect[39,40] or no effect[41,42]. In the present study, most of the MMT patients experienced adverse traumatic events (S1 Table), such as lower education levels, a broken family, unemployment and divorce. Stressful events (e.g., unemployment, adverse family dynamics and divorce) might increase cortisol secretion, although the use of the education level as a covariate had no significant influence on the hair cortisol level. Third, there might be other underlying biological mechanisms. One straightforward potential explanation for the relatively high cortisol level in the MMT patients was the abnormal decrease in cortisol metabolism (e.g., cortisol metabolizes as cortisone). Patients undergoing long-term MMT may develop a metabolic dysfunction or disorder because methadone gives rise to dysfunction of the HPA axis [1]. The disorder may lead to a series of physical, psychological and pathological changes. For example, a previous study found that MMT patients showed reduced drug cravings and withdrawal symptoms at the beginning of the methadone treatment, but no longer felt any significant physical and psychological progress even if the MMT was continued[43]. These above-mentioned explanations need to be confirmed in future research.

There were some limitations in the present study. First, this study only recruited male patients, which limited the extension of the conclusion. Second, this study only recruited age-matched healthy participants as controls and did not recruit heroin addicts without MMT as controls. Therefore, this study did not provide firm evidence to support whether methadone suppressed the hair cortisol level or the function of the HPA axis. Third, this study did not measure the score of drug craving. Although methadone attenuates heroin craving, drug cue-related stimuli possibly triggers craving and stress arousal in methadone-maintained patients after methadone intake [16]. In this study, methadone patients must continue to take methadone might be a drug cue, thereby inducing higher drug craving and stress and resulting in the elevated hair cortisol in the present MMT patients compared to controls and even heroin addicts who showed lower drug craving than MMT patients[4,15]. Fourth, this study did not control for the possible effect of the employment status, early family condition and marital status on hair cortisol levels because it did not investigate the early family condition and marital status in the controls. Additionally, this study did not obtain information concerning previous traumatization in the MMT patients, such as a history of childhood trauma. Fourth, this investigation did not collect information on the frequency of hair washing, which might affect the hair cortisol level.

## Conclusion

Hair cortisol level of MMT patients is an endogenous biomarker of the basal activity of the HPA axis and stress state as demonstrated by significant association between hair cortisol and cortisol in overnight urine. Long-term MMT patients showed significantly more symptoms of depression and anxiety and significantly higher perceived stress levels than non-heroin-dependent controls. They also showed significantly higher hair cortisol levels than the controls, indicating that patients with long-term MMT might show higher basal activity of the HPA axis. However, the difference in hair cortisol was not significant when the perceived stress, depression and anxiety scores were used as covariates, indicating that the high chronic stress state and increase in depression and anxiety symptoms may mask the suppression effect of methadone on the HPA activity.

## Supporting Information

S1 Dataset Data of the MMT patients and controls. (SAV)

S1 Table Demographic data and psychological states of the MMT patients and controls, the durations of heroin consumption and methadone administration, and the employment status of the MMT patients. (DOCX)

S2 Table Regression coefficients of log-transformed hair cortisol levels against the durations of MMT administration and heroin consumption among the MTT patients. (DOCX)
