# Supplementary material for: Elevated Hair Cortisol Levels among Heroin Addicts on Current Methadone Maintenance Compared to Controls
Source: PLoS One. 2016 Mar 24;11(3):e0150729. doi: 10.1371/journal.pone.0150729 (PMC4806835; doi:10.1371/journal.pone.0150729)
Supplement: S1 Table — (DOCX) [file pone.0150729.s002.docx]

S1 Table: Demographic data and psychological states of the MMT patients and controls, the durations of heroin consumption and methadone administration, and the employment status of the MMT patients.

|  | MMT group (*n*=52) | Control (*n*=41) | Statistical value ^a^ |
| --- | --- | --- | --- |
| Age |  | |  |
| *M*±*SD* (years) | 40.6±6.2 | 42.2±12.2 | *t*_56.20_=-0.738 |
| Range (years) | 28~56 | 22~61 |  |
| Education level: number (%) | | | *χ*^2^=26.852*** |
| elementary school and below (EL1) | 5 (9.6) | 2 (4.9) |  |
| junior high school (EL2) | 29 (55.8) | 6 (14.6) |  |
| senior high school (EL3) | 17 (32.7) | 19 (46.3) |  |
| college and university and over (EL4) | 1 (1.9) | 14 (32.1) |  |
| Employment state: number (%) | | | |
| employment | 11 (21.1) | − |  |
| unemployment | 33 (63.5) | − |  |
| N/A | 8 (15.4) | − |  |
| Family condition when he/she was younger than 18 years old: number (%) | | | |
| A whole family | 33 (63.5) | − |  |
| A broken family (divorced parents, death of parents or sibling) | 19 (36.5) | − |  |
| Marital status: number (%) |  |  |  |
| single | 5 (9.6) | − |  |
| married | 27 (51.9) | − |  |
| divorced | 20 (38.5) | − |  |
| Duration of heroin consumption (M±SD) (years) ^b^ | 13.4±3.3 | − |  |
| Duration of methadone maintenance treatment (M±SD) (months) ^b^ | 33.8±26.0 | − |  |
| Psychological characteristics |  | | |
| SDS score (*M*±*SD*) | 48.0±9.2 | 33.1±7.4 | *t*_91_=8.399*** |
| SAS scores (*M*±*SD*) | 43.2±8.7 | 29.5±6.5 | *t*_91_=8.470*** |
| Prevalence of depression: number (%) ^c^ | | | *χ*^2^=41.917*** |
| 40～47 | 16 (30.8) | 6 (14.6) |  |
| 48～55 | 17 (32.7) | 2 (4.9) |  |
| >56 | 11 (21.2) | 0 (0) |  |
| Prevalence of anxiety: number (%) ^d^ | | | *χ*^2^=31.366*** |
| 40～47 | 16 (30.8) | 3 (7.3) |  |
| 48～55 | 13 (25.0) | 0 (0) |  |
| >56 | 4 (7.7) | 0 (0) |  |

Notes: ^*^ *p*<0.05, ^**^ *p*<0.01, ^***^ *p*<0.001. *M* was the mean and *SD* was the standard deviation. ^a^ *t* was the statistical value of the *t*-test for two independent samples and *χ*^2^ was the value of the Chi-square test. ^b^ One MMT patient did not provide the durations of heroin consumption and methadone administration. ^c^ A raw SDS score of 40 was used as the depression cutoff score. ^d^ A raw SAS score of 40 was used as the anxiety cutoff score.
