# Supplementary material for: Elevated Hair Cortisol Levels among Heroin Addicts on Current Methadone Maintenance Compared to Controls
Source: PLoS One. 2016 Mar 24;11(3):e0150729. doi: 10.1371/journal.pone.0150729 (PMC4806835; doi:10.1371/journal.pone.0150729)
Supplement: S2 Table — (DOCX) [file pone.0150729.s003.docx]

S2 Table: Regression coefficients of log-transformed hair cortisol levels against the durations of MMT administration and heroin consumption among the MTT patients

|  | B | SE | *β* | p |
| --- | --- | --- | --- | --- |
| Level 1 |  |  |  |  |
| age | 0.008 | 0.009 | 0.133 | 0.378 |
| PSS | -0.005 | 0.011 | -0.084 | 0.657 |
| SDS | -0.006 | 0.010 | -0.137 | 0.539 |
| SAS | 0.004 | 0.009 | 0.086 | 0.653 |
| Level 2 |  |  |  |  |
| duration of heroin consumption | 0.00009 | 0.020 | 0.001 | 0.996 |
| duration of MMT administration | -0.004 | 0.002 | -0.269 | 0.084 |

Notes: B is the unstandardized regression coefficient, SE is standard error of the mean, and *β* is the standardized regression coefficient.
